# Mental health status and coping strategies during COVID-19 pandemic among university students in Central Serbia

**DOI:** 10.3389/fpsyt.2023.1226836

**Published:** 2023-10-25

**Authors:** Jovana Radovanovic, Viktor Selakovic, Olgica Mihaljevic, Jelena Djordjevic, Sofija Čolović, Jelena R. Djordjevic, Vladimir Janjic, Milena Jovicic, Sladjana Veselinovic, Ivana Simic Vukomanovic, Katarina Janicijevic, Zivana Slovic, Snezana Radovanovic, Ognjen Djordjevic, Milan Djordjic

**Affiliations:** ^1^Faculty of Medical Sciences, University of Kragujevac, Kragujevac, Serbia; ^2^Department of Pathophysiology, Faculty of Medical Sciences, University of Kragujevac, Kragujevac, Serbia; ^3^Department of Communication Skills, Ethics, and Psychology, Faculty of Medical Sciences, University Kragujevac, Kragujevac, Serbia; ^4^University Clinical Centre Kragujevac, Clinic for Psychiatry, Kragujevac, Serbia; ^5^Department of Psychiatry, Faculty of Medical Sciences, University of Kragujevac, Kragujevac, Serbia; ^6^Center for Harm Reduction of Biological and Chemical Hazards, Faculty of Medical Sciences, University of Kragujevac, Kragujevac, Serbia; ^7^Department of Psychology, Faculty of Medical Sciences, University of Kragujevac, Kragujevac, Serbia; ^8^Department of Social Medicine, Faculty of Medical Sciences, University of Kragujevac, Kragujevac, Serbia; ^9^Department of Forensic Medicine, Faculty of Medical Sciences, University of Kragujevac, Kragujevac, Serbia; ^10^University Clinical Centre Kragujevac, Forensic Medicine and Toxicology Service, Kragujevac, Serbia; ^11^Department of Epidemiology, Faculty of Medical Sciences, University of Kragujevac, Kragujevac, Serbia

**Keywords:** anxiety, Central Serbia, COVID-19 pandemic, coping strategies, depression, stress, university students

## Abstract

**Background:**

The objective of this research was to evaluate the occurrence of depression, anxiety, and stress, as well as the level of life contentment and coping mechanisms employed by college students amidst the COVID-19 pandemic.

**Methods:**

A total of 588 students of the Faculty of Mechanical Engineering and the Higher School of Medicine, Kraljevo, University of Kragujevac, Central Serbia, participated in an online cross-sectional survey in the period September–October 2022. Depression Anxiety Stress Scales (DASS-21) was used to measure the levels of depression, anxiety, and stress. The Coping Questionnaire—a shorter version (Brief Cope Inventories) assesses the coping strategies a person uses in stressful situations, and the 5-item Life Satisfaction Scale is used to examine the level of life satisfaction.

**Results:**

The symptoms of depression were reported by 34.9%, of anxiety by 47.1% and of stress by 44.2%. The type of faculty (*p* = 0.001), and place of residence (*p* = 0.036) correlated with depression, gender with anxiety (*p* = 0.001) and stress (*p* = 0.015). In terms of coping mechanisms, the most frequently mentioned strategies, based on average scores, were acceptance (5.76), positive interpretation (5.55), humor (5.46).

**Conclusion:**

The adverse impact of COVID-19 was a risk factor for depression, anxiety and stress symptoms. A negative correlation exists between the levels of depression, anxiety, and stress, and the overall life satisfaction of university students. Offering the necessary assistance through psychological interventions and effective coping techniques is crucial in ensuring the optimal mental health of university students.

## Introduction

The COVID-19 pandemic has emerged as a significant global public health crisis, posing substantial challenges to mental wellbeing. Student mental health has become a focal point of research during this pandemic, with numerous studies conducted worldwide shedding light on various aspects of mental health. These studies have consistently highlighted a decline in student mental health, with varying degrees of mental disorders, primarily anxiety, stress, and depression (1–13). The closure of educational institutions has brought about drastic changes in students’ daily routines. In-person classes have been replaced by online sessions, resulting in reduced social interactions among students and an upsurge in stress, anxiety, and depressive symptoms within this population (14, 15).

Research findings have consistently indicated a notable occurrence of stress, anxiety, and depression among students amid the COVID-19 pandemic, stemming from the consequences of the disease itself as well as the accompanying containment measures. Diverse sources of stress were reported, encompassing disruptions in educational engagements, financial adversities, prolonged shutdowns of educational institutions, overwhelming exposure to COVID-19-related information, the shift to homeschooling, apprehension regarding virus transmission, and the psychological effects of stringent measures like quarantine, isolation, and physical distancing (16–22). College students, in particular, exhibited elevated levels of mental distress, indicating the detrimental effects of the ongoing pandemic on their psychological wellbeing (23). The findings of several studies conducted in Serbia testify to that. The study was conducted during the increased incidence of COVID-19 and the mean score of perceived stress amounted to 20.43 (± 7.67) (24). Another study was during the school year 2020–2021. It used Depression Anxiety Stress Scales (DASS-21) and showed that two thirds of the students who participated (age ranging from 21 to 30) reported symptoms of depression, extremely severe forms of anxiety, and severe stress (25).

However, research into the mental health of students before the pandemic onset clearly suggests a notable impact of the pandemic on students’ mental wellbeing. Reported levels of depression, anxiety, and stress were considerably lower compared to studies conducted during the pandemic period. So, cross-sectional survey of students attending University of Kragujevac, Serbia, before the pandemic indicate that depression and anxiety were present in 23.6 and 33.5% of students (26). Therefore, the stress and limitations linked to the COVID-19 pandemic place students in a vulnerable position, increasing their susceptibility to developing mental health issues that can significantly impact their academic performance, social interactions, and future professional and personal prospects (23).

Within this context, the aim of this study was to identify sociodemographic variables associated with depressive, anxiety and stress symptoms among university students during pandemic COVID-19, as well as the level of life contentment amidst the COVID-19 pandemic. Also, the study aims to shed light on the coping strategies employed by students in their psychological battle against the virus.

The global impact of the COVID-19 pandemic on students is a matter of widespread concern. Identifying various determinants of mental health may hold particular significance in formulating public health interventions and implementing necessary approaches to enhance mental wellbeing and prevent mental disorders during the COVID-19 pandemic, as well as in preparing for unforeseen pandemics or disasters in the future.

## Methods

### Design

A total of 588 students of the Faculty of Mechanical Engineering and the Higher School of Medicine, Kraljevo, University of Kragujevac, Central Serbia, participated in an online cross-sectional survey in the period September–October 2022.

### Sample

Random sampling was used as the sample selection method. The 10-min survey was conducted electronically. It was completely voluntary and anonymous. The sample included students from all years of study and of both genders. The response rate was 86%. Inclusion criteria for study participants are as follows: students of the Faculty of Mechanical Engineering and the Higher School of Medicine, Kraljevo, University of Kragujevac, aged 19 and above, of both genders, without a previously diagnosed mental disorder, and having obtained written consent to participate in the study.

Exclusion criteria from the study are: students from other faculties, under the age of 19, diagnosed with a mental disorder, incompletely filled questionnaires, and students from whom written consent to participate in the study has not been obtained.

### Ethical considerations

The respondents provided informed consent after they had been informed about the methodology and the purpose of this study on the first page of the electronic platform used to conduct the survey. The data were treated as highly confidential and were used for research purposes only. The questions that might identify the respondents were avoided. All necessary steps were taken, in accordance with the General Regulation for the Protection of Personal Data, the legislation of the Republic of Serbia, the European Legal Framework, the National Data Protection Act, the Strategy for the Protection of Personal Data, and the Law on Official Statistics Act, in order to protect the privacy and ensure the confidentiality of the data.

Deans of the Faculty of Mechanical Engineering and the Higher Medical School, Kraljevo, University of Kragujevac, Central Serbia, gave written consents for the survey to be conducted.

### Surveys

The research instruments were linguistically and culturally validated questionnaires in the Serbian language. General Questionnaire was used to collect demographic and personal data about students’ lives before and during the COVID-19 pandemic. It was used to collect data on gender, age, type of settlement, faculty, and year of study. In addition, it included questions inquiring whether the students felt endangered during the pandemic and what the reasons were for them to feel threatened: the fear of getting infected, the fear of endangering family members, the fear that close people (family, friends, etc.) may get infected, the fear of hospitalization, etc.

The standardized Serbian version of Depression Anxiety Stress Scales (DASS-21) was used to measure the levels of depression, anxiety, and stress. The Psychometric evaluation of the Depression, Anxiety, and Stress Scale-21 (DASS-21) was conducted on a sample of 1,374 students from the University of Novi Sad in Serbia (27). The Serbian version of this questionnaire is publicly available at: https://www2.psy.unsw.edu.au/dass/Serbian/DASS-CYR.pdf.

The questionnaire consists of 21 questions and three subscales which aim at evaluating the levels of depression, anxiety, and stress. Respondents rated on a 4-point Likert scale how they felt during the past week, i.e., how strongly/frequently they experienced symptoms of depression, anxiety, and stress, from 0 (“not at all”) to 3 (“most of the time or almost always”). Depression, anxiety, and stress scores were obtained by summing the scores of the corresponding items in the range of 0–21 for each subscale. Symptom severity was graded using cut-off values to define normal, mild, moderate, significant, and very significant values for each subscale. For the “Depression” scale, a total score of 0–4 normal; 5–6 mild depression; 7–10 moderate depression; 11–13 severe depression; ≥ 14 very severe depression. For the “Anxiety” scale, a score of 0–3 is considered normal; 4–5 mild anxiety; 6–7 moderate anxiety; 8–9 severe anxiety; ≥ 10 very severe anxiety. For the “Stress” scale, a score of 0–7 is considered normal; 8–9 mild stress; 10–12 moderate stress; 13–16 severe stress; ≥ 17 very severe stress. Very serious symptomatology is defined by a depression subscale score of 14+, anxiety of 10+, and stress of 17+. The scores given indicate the severity of symptoms, not the degree of mental disorder (27).

Coping Questionnaire—a shorter version (Brief Cope Inventory) assesses the coping strategies a person uses in stressful situations (15). The questionnaire contains 28 questions assessing 14 strategies: distraction (items 1 and 19), active coping (items 2 and 7), denial (items 3 and 8), substance use (items 4 and 11), emotional support (items 5 and 15), instrumental support (items 10 and 23), Behavioral withdrawal (items 6 and 16), venting (items 9 and 21), positive reinterpretation (items 12 and 17), planning (items 14 and 25), humor (items 18 and 28), acceptance (items 20 and 24), religion (items 22 and 27), self-blame (items 13 and 26). Respondents answered on a four-point Likert scale ranging from 1 (“I do not use at all”) to 4 (“I use often”). The total subscale scores are the sum of the scores for the individual items within each subscale. On each of the 14 subscales, a higher mean score indicates that a particular coping strategy is used more often (28).

The Life Satisfaction Scale (The 5-item Life Satisfaction Scale) is used to examine the level of life satisfaction of students, in general, using five self-assessment questions on a seven-point scale. The scale consists of 5 statements and the respondent rates their level of agreement with each statement on a seven-point Likert scale (7—“strongly agree,” 6—“agree,” 5—“somewhat agree,” 4—“neither agree nor disagree,” 3—“somewhat disagree,” 2—“disagree,” 1—disagree at all”). Scores can range from 5 to 35 (5–9 extremely dissatisfied, 10–14 dissatisfied, 15–19 somewhat dissatisfied, 20 neutral, 21–25 slightly satisfied, 26–30 satisfied, 31–35 extremely satisfied), with higher scores indicating a higher degree of satisfaction with one’s life. The Life Satisfaction Scale measures the cognitive component of personal wellbeing. The authors state that the reliability of the questionnaire is 0.85 (29).

The variables taken into consideration here include (1) sociodemographic characteristics (gender, age, type of settlement, type of faculty, year of study), (2) characteristics of life and study during the pandemic COVID-19, (3) Mental health: i.e., depression, anxiety, stress, (4) coping mechanisms: distraction, active coping, denial, substance use, emotional support, instrumental support, behavioral withdrawal, venting, positive reinterpretation, planning, humor, acceptance, religion, self-blame; (5) satisfaction with life.

### Statistical analysis

All statistical calculations were performed with the standard commercial software package SPSS, version 18.0. [Statistical Package for the Social Sciences software (SPSS Inc., version 18.0, Chicago, IL)]. Data cleaning was done to detect any missing values, coding error or any illogical data values. The qualitative variables (demographic and socioeconomic) were presented with the numbers and as a percentage. The continuous variables (depression, anxiety, and symptoms scores), were presented as means and standard deviation (SD). Descriptive statistics for all sociodemographic characteristics, depressive and anxiety symptoms of the participants were calculated, expressed as appropriately in frequencies, mean values and standard deviation. Chi-square (χ^2^) test was used to compare differences in frequencies of categorical variables. The magnitude of the connection between observed phenomena was determined through correlation analysis. All results with a probability of less than 5% (*p* < 0.05) were considered statistically significant.

## Results

The sociodemographic characteristics of the student population are shown in Table 1.

**Table 1:** Sociodemographic characteristics and the proportion of university students with symptoms of depression, anxiety and stress.

Variables	Total		Depression		*p*	Anxiety		*p*	Stress		*p*
	n	%	n	%		n	%		n	%	
Total	493	100.0	172	34.9		232	38.4		218	40.7	
Gender											
Male	115	23.3	41	35.6	0.985	42	36.4	0.001	39	34.0	0.015
Female	378	76.7	131	34.7		190	40.4		179	47.4	
Age groups											
18–24	331	67.1	124	37.6	0.132	158	47.8	0.383	163	49.3	0.015
25–29	42	8.5	14	33.4		21	50.0		14	33.3	
30–34	30	6.1	10	33.3		15	50.1		8	26.7	
35–39	35	7.1	9	45.7		13	37.2		14	40.0	
40±	55	11.2	15	27.2		25	45.5		19	34.5	
Type of settlement											
Urban	349	70.8	111	31.8	0.036	157	44.9	0.390	150	43.0	0.130
Rural	144	29.2	61	32.3		75	52.1		68	47.2	
Type of faculty											
Faculty of Mechanical Engineering	82	16.6	42	51.2	0.001	38	46.3	0.887	40	48.8	0.362
Higher School of Medicine	411	83.4	130	31.6		194	47.2		178	43.3	
Year of study											
First year	171	34.7	56	32.7	0.454	86	50.3	0.766	17	9.9	0.408
Second year	140	28.4	52	37.1		59	42.1		20	14.3	
Third year	163	33.1	55	33.7		79	48.4		12	74.0	
Fourth year	11	2.2	7	63.7		6	54.6		0	0.0	
Fifth year	8	1.6	2	25.0		2	25.0		1	12.5	

Studija je sprovedenana 493 students, 16.6% of which were from the Faculty of Mechanical and Civil Engineering and 76.7% of which were from the Higher School of Medicine. 23.3% of the respondents were male and 76.7% female. The average age was 25.4 ± 8.3 years.

### Depression

It was determined that depression symptoms were present in 34.9% of students, distributed as follows: 10.5% experiencing mild, 11.6% moderate, 6.9% severe, and 5.9% very severe levels of depression. The mean depression score was 4.32 ± 4.67. An analysis of the impact of sociodemographic variables on the severity of depression among students during the COVID-19 pandemic indicated that there were no significant differences in depression based on gender (*p* = 0.985), age groups (*p* = 0.132), years of study (*p* = 0.454), but there was a significant difference based on the type of faculty (*p* = 0.001), and place of residence (*p* = 0.036). Specifically, students of the Faculty of Mechanical Engineering exhibited 1.6 times higher levels of depression (51.2%) compared to students from the Higher School of Medicine (31.6%). Furthermore, students from rural areas displayed slightly higher levels of depression symptoms than students from urban areas (32.3% vs. 31.8%) (Table 1).

### Anxiety

Anxiety symptoms were reported by 38.4% of students, distributed as follows: 14.8% with mild, 10.5% with moderate, 10.3% with severe, and 11.4% with very severe levels of anxiety. The mean anxiety score was 4.57 ± 4.45. Gender significantly correlated with anxiety (*p* = 0.001), with females exhibiting symptoms of anxiety more frequently than males (40.4% vs. 36.4%). However, other sociodemographic factors did not influence the manifestation of anxiety symptoms (Table 1).

### Stress

Reported by 40.7% of students, the manifestations of stress were distributed as such: 10.1% with mild, 13.8% with moderate, 12.8% with severe, and 7.5% with exceedingly severe degrees of stress. The mean score for stress reached its pinnacle at 8.02 ± 5.15. The findings underscore a noteworthy divergence in outcomes between male and female participants (*p* = 0.015). Females exhibited stress symptoms at a 1.3-fold higher rate than males (47.4% vs. 34%). The incidence of stress among students diminishes as they progress in age (p = 0.015). Notably discernible variations in the prevalence of stress among students were absent concerning the distinction between urban and rural dwellings (*p* = 0.130), academic progression (*p* = 0.408), or academic discipline (*p* = 0.362) (Table 1).

### Life satisfaction

Applying the Life Satisfaction Scale, we established that the largest percentage of the studied student population belongs to the life satisfaction category (74.6%), while every fifth student expressed dissatisfaction with life (19.4%). The largest percentage of strongly concur that their living conditions are exceptional (19.7%) and express contentment with their lives (17.6%). Conversely, the greatest percentage express disagreement with the assertion that they would not alter anything in their lives (11.8%) (Table 2).

**Table 2:** Life satisfaction of the university students population.

Life satisfaction	1*	2	3	4	5	6	7
My life is almost ideal	4.1	7.9	8.3	28.8	29.0	16.4	5.3
My living conditions are excellent	1.6	2.8	5.1	17.0	29.2	24.3	19.7
I am satisfied with my life	2.2	1.8	5.9	14.4	27.4	30.4	17.6
So far I have achieved the important things I want in life	2.4	7.1	7.5	17.8	29.4	22.3	13.0
If I were born again, I would not change anything	11.8	11.0	8.1	20.5	15.0	17.2	16.2
Life satisfaction scores**	5–9 (1.8%)	10–14 (5.5%)	15–19(12.4%)	20 (5.7%)	21–25 (31.8%)	26–30 (29.4%)	31–35 (13.4%)

*1, I do not agree at all; 2, I do not agree: 3, I disagree for the most part; 4, I neither agree nor disagree; 5, I agree for the most part; 6, I agree; 7, I agree completely. **Life satisfaction scores: (5–9 extremely dissatisfied, 10–14 dissatisfied, 15–19 somewhat dissatisfied, 20 neutral, 21–25 slightly satisfied, 26–30 satisfied, 31–35 extremely satisfied).

**GRAPH 1:**
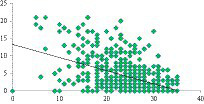
Correlation between depression and life satisfaction.

**GRAPH 2:**
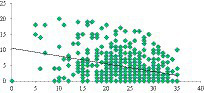
Correlation between anxiety and life satisfaction.

**GRAPH 3:**
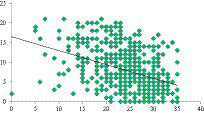
Correlation between stress and life satisfaction.

A significant negative correlation is observed between the total depression score and the life satisfaction scale (*r* = −0.483, *p* = 0.000). Students who reported lower levels of life satisfaction demonstrated higher levels of depression, and vice versa (Graph 1). The correlation analysis further demonstrates an inverse association between the level of anxiety and students’ life satisfaction (*r* = −0.483, *p* = 0.000) (Graph 2). Additionally, the correlation analysis reveals an inverse relationship between the level of life satisfaction and the level of stress experienced by students (*r* = −0.483, *p* = 0.000). Students who expressed higher levels of life satisfaction experienced lower levels of stress, and vice versa (Graph 3).

### Coping strategies

Table 3 showcases the distribution of coping strategies based on the Brief Cope Inventory. Students predominantly utilized positive coping mechanisms. The prevailing strategy among students is embracing reality (42.7%), closely followed by seeking something good in what has happened (38.3%), engaging in leisure activities such as going to the cinema, watching TV, reading, daydreaming, and shopping (32.9%), trying to think of strategies on what to do (29.8%), taking action to improve the situation (29.1%), making jokes about the problem (28.7%), and accepting help from others (27.6%). Negative coping mechanisms were much less common. 3.2% of students reported using alcohol or other substances to feel better.

**Table 3:** The distribution of coping strategies based on Brief Cope Inventory.

Coping strategies	Not at all	Little	Moderately	Often
I turn to work or other activities to distract me from the problem	27.6	28.2	21.3	22.9
I make an effort to do something about the situation I am in	25.4	26.4	30.4	17.8
I ask myself, “Is this really happening to me?”	47.1	28.4	13.2	11.4
I consume alcohol or other substances to make myself feel better	83.4	9.3	4.1	3.2
I get emotional support from others	19.1	26.4	28.8	25.8
I give up trying to cope with the problem	76.1	15.0	5.5	3.4
I take action to try to improve the situation	18.7	23.6	28.5	29.1
I refuse to believe that this is happening to me	63.8	22.0	9.3	4.9
I talk to myself to get rid of negative feelings	51.7	20.9	14.6	12.8
I accept help and advice from others	11.6	26.8	33.9	27.6
I take alcohol or other substances to cope with this situation	88.8	5.9	2.4	2.8
I try to see the situation in a different light, to find a positive alternative	22.8	20.9	29.1	27.2
I criticize myself	31.8	29.4	21.3	17.4
I’m trying to make a strategy for what to do	17.8	26.0	26.4	29.8
I get consolation and understanding from others	19.3	30.6	27.2	22.9
I give up trying to deal with the problem	77.0	14.6	5.1	3.3
I look for something good in what has happened	12.8	18.3	30.4	38.5
I make jokes about the problem	14.9	19.1	27.3	28.7
I engage in activities such as going to the cinema, watching TV, reading, daydreaming, sleeping, shopping…	18.9	21.7	26.6	32.9
I accept the reality and the fact that it happened	14.0	14.4	28.9	42.7
I express my negative feelings	28.0	36.2	19.3	16.5
I try to find comfort in religion or spiritual beliefs	46.3	26.8	14.4	13.2
try to take advice or help from others about what to do	18.1	29.1	33.1	19.7
I learn to live with my problems	15.4	23.5	30.0	31.0
I have a hard time thinking about what steps to take	46.0	29.6	15.0	9.3
I blame myself for the things that have happened	55.0	25.4	11.4	8.3
I pray or meditate	51.7	22.1	12.0	14.2
I make jokes about this situation	24.3	23.7	21.9	30.0

Based on mean coping strategy scores, the most commonly reported coping strategies were acceptance (5.76), positive interpretation (5.55), humor (5.46), instrumental support (5.31), and emotional support (5.15). The lowest mean scores were recorded for behavioral withdrawal (2.71) and substance use (2.46) (Table 4).

**Table 4:** Coping strategies scores in the university student population.

Coping strategies	Mean	SD
Distraction	5.13	1.82
Active coping	5.08	1.89
Denial	3.44	1.61
Substance use	2.46	1.24
Emotional support	5.15	1.89
Instrumental support	5.31	1.78
Behavioral withdrawal	2.71	1.34
Venting	4.12	1.66
Positive reinterpretation	5.55	1.91
Planning	4.56	1.64
Humor	5.46	2.11
Acceptance	5.76	1.83
Religion	3.83	1.96
Self-blame	3.97	1.81

## Discussion

The results of this research showed that students report symptoms of depression in 34.9%, anxiety in 47.1% and stress in 44.2%. Although our study was conducted 2 years after the onset of the pandemic, the findings are alarming. However, if these results are compared to the findings of one study conducted at the beginning of the pandemic, we can observe that levels of stress, depression, and anxiety declined. Reportedly, at the onset of the pandemic, 64.5% of students reported the symptoms of depression, 66.8% severe levels of anxiety, and 66.7% extremely severe symptoms of stress (24). These results may suggest that students adapted to new circumstances and developed coping mechanisms.

Similar to our study, other research has identified that almost 45% of students faced mental health issues, with anxiety being the most frequent symptom (3). In a sample of 1,173 undergraduate and postgraduate students from a university in the UK, over 50% of respondents surpassed clinical thresholds for anxiety and depression (21).

Likewise, a cross-national study involving college students from nine countries documented a significant occurrence of stress (61.3%), depression (40.3%), and anxiety (30%) (4). Additional studies have also indicated that 48.14% of college students reported moderate to severe depression, 38.48% experienced mild to severe forms of anxiety, and 18.04% reported having suicidal thoughts during the COVID-19 pandemic (30).

And the results of our study are concerning, as a notable 12.8% of students exhibited severe and extremely severe signs of depression, while 21.7% experienced severe and extremely severe levels of anxiety, and 20.3% reported severe and extremely severe stress conditions. Furthermore, our findings demonstrated that there is no significant difference in depression between male and female participants, whereas a distinction exists when it comes to anxiety and stress, where female students displayed higher symptoms of anxiety and stress compared to their male counterpart. And other studies showed that female participants had higher scores on the PHQ-9 and GAD-7 scales, while male respondents in higher academic years had lower results (30).

Also, depression was more prevalent among participants from rural areas and among students of engineering disciplines. The occurrence of stress among students diminishes with increasing age. Other studies have also demonstrated similar findings, where age was identified as a protective factor against stress (31).

Research findings indicate that there were no significant variations in the prevalence of depression, anxiety, and stress among students concerning their year of study. Despite having established that two-thirds of the surveyed student population falls under the category of life satisfaction, there exists, a negative correlation exists between the levels of depression, anxiety, and stress, and the overall life satisfaction of university students. Furthermore, we determined that students largely utilized positive coping mechanisms, with the prevailing strategy among students being acceptance of reality. Other studies have also focused on examining how students cope with stress during the pandemic. A study conducted in China, involving 7,800 students, revealed that resilience, adaptive coping strategies, and social support played a mediating role in the relationship between negative experiences with COVID-19 and acute stress disorder (32). Similarly, another study identified that college students employed video chatting, social media, sports, and new hobbies as coping mechanisms during the pandemic (20). Correspondingly, a qualitative study conducted among international students enrolled in a UK college reached similar conclusions, with students reporting activities such as watching movies, engaging in conversations with friends and family, and participating in exercise during the pandemic (32). Additionally, alongside positive coping strategies, some studies have highlighted the utilization of negative coping strategies, including smoking and alcohol consumption, by students (30, 33, 34).

Son et al. conducted a study examining students’ coping strategies for managing stress and anxiety associated with illness, particularly in relation to COVID-19. Their findings revealed that individuals adopted various coping methods, such as seeking support from others, utilizing positive techniques like meditation, breathing exercises, and spiritual approaches, as well as employing negative approaches such as ignoring COVID-related news. The study highlighted that passive coping strategies, excessive exposure to pandemic information, and a lack of social support from a partner were factors contributing to heightened levels of psychological distress within the Chinese community during the outbreak (23).

Regarding the question about coping mechanisms used to alleviate stress and anxiety, a significant proportion of students (67.06%) indicated seeking support from their community, family, and friends as their primary approach. Technology-based tools (websites, mobile apps, health monitoring sensors) were also mentioned by a substantial percentage of respondents (32.45%). A smaller number of students reported utilizing university counseling services (10.34%) or external health services (4.38%). Many participants (39.0%) emphasized adopting a healthy lifestyle, including engaging in regular exercise, maintaining a proper diet, and practicing self-care activities. Similarly, a considerable portion of respondents (37.1%) reported engaging in relaxation techniques such as meditation, reading, playing with pets, listening to music, breathing exercises, sleep, hobbies like gardening, and other general relaxation activities. Additionally, some participants (8.0%) expressed involvement in creative endeavors, such as artwork creation, writing, and playing musical instruments. Furthermore, respondents highlighted their engagement in spiritual and religious practices. A small percentage of individuals (10.6%) acknowledged resorting to negative coping methods such as excessive drinking, self-isolation, self-harm, and crying (35). Notably, a portion of the participants (19.05%) indicated that they did not utilize any specific coping mechanisms.

Our research results shed light on the strategies employed by students to navigate the mental health challenges brought about by the pandemic. The majority of students demonstrated resilience by utilizing various coping mechanisms and seeking support from different sources. Among the most commonly reported coping strategies were acceptance, positive reinterpretation of situations, humor, instrumental support (practical assistance), and emotional support. On the other hand, behavioral withdrawal, characterized by avoidance or retreat, was identified as the least frequently employed coping mechanism among students. These findings underscore the adaptive nature of students in managing their mental wellbeing during challenging times.

A comprehensive study conducted in Nepal examined the stress levels and sources of stress among college students during the pandemic-induced full closure in July 2020. The study encompassed 615 students and revealed significant variations in perceived stress levels based on factors such as student age, majors and levels of study, living status (whether residing with or outside the family), parent’s occupation, and family income. Regarding coping strategies, the mean score indicated that the most commonly utilized coping mechanism among students was self-distraction (with a score of 3.3 ± 0.9), while substance use was found to be the least preferred coping strategy (with a score of 1.2 ± 0.5). These findings highlight the diverse coping strategies adopted by college students in Nepal to manage stress during the pandemic. The study underscores the importance of understanding individual differences in stress levels and the subsequent selection of coping strategies for effective support and intervention in promoting students’ mental wellbeing (36).

The significance of our study lies in its exploration of the pandemic’s impact on levels of stress, anxiety, and depression, as well as their correlation with sociodemographic factors. Moreover, our study aims to shed light on the coping strategies employed by students during the pandemic. Deliberately, we selected one medical and one non-medical educational profile, expecting that students in the health field might be better equipped to cope in their psychological battle against the virus. A particular emphasis is placed on the fact that the research was conducted during the later stages of the pandemic. Therefore, the results have the potential to offer insights into the evolving dynamics throughout different phases of the pandemic.

However, our study has certain limitations related to the coverage of the student population. Students of different profiles and from different regions of Serbia should be included, which can be the subject of a subsequent study.

## Conclusion

The results of this study testify to the fact mental health of the youth should be monitored through consistent and comprehensive research. Based on the findings of our study, it is recommended to implement stress management and life skills training programs for students. These programs can provide valuable tools and strategies to help students effectively cope with stress and enhance their overall wellbeing. Additionally, further research is warranted to explore the long-term consequences of the pandemic on students’ mental health, as it can inform the development of targeted interventions and support initiatives. In terms of policy, it is crucial to prioritize promotion and prevention measures in the field of student mental health. This entails providing accessible psychological support services outside of traditional healthcare settings, such as schools, dormitories, and boarding schools. Establishing specialized mental health counseling centers in community settings can ensure that students have easy access to the support they need. Primary prevention efforts should also be emphasized, both through the educational system and various forms of mass communication, to raise awareness and promote mental health literacy.

These findings can improve our future preparedness in case of other unexpected pandemic or disaster. Viewing mental health promotion and the prevention of mental disorders among students as public health priorities is vital for the overall progress and stability of society.

## Data availability statement

The original contributions presented in the study are included in the article/supplementary material, further inquiries can be directed to the corresponding author.

## Ethics statement

The studies involving humans were approved by the Deans of the Faculty of Mechanical Engineering and the Higher Medical School, Kraljevo, University of Kragujevac, Central Serbia, gave written consents for the survey to be conducted. The studies were conducted in accordance with the local legislation and institutional requirements. The participants provided their written informed consent to participate in this study.

## Author contributions

JR, VS, JD, SC, and VJ design and writing manuscript. OM, SR, SV, and MJ conducted the statistical analyses, commented on the manuscript and contributed to the background, and discussion section. ISV and KJ contributed to investigation. JRD, ZS, OD, and MD critical revision and approval of the final draft. All authors contributed to the article and approved the submitted version.
